# Terms of Pupilage

**Published:** 1883-08

**Authors:** 


### TERMS OF PUPILAGE.

The number of years a medical student has to spend at a medical institution prior to being admitted to examination for a medical degree, in various countries, is as follows: Sweden, ten; Holland, Italy, and Switzerland, six; Norway, eight; Denmark, six and seven; Belgium, six; Russia, Portugal, Austria and Hungary, five; France, England, and Canada, four; United States, three or two; Spain, two.

